# Imaging systems and algorithms to analyze biological samples in real-time using mobile phone microscopy

**DOI:** 10.1371/journal.pone.0193797

**Published:** 2018-03-06

**Authors:** Akshaya Shanmugam, Mohammad Usmani, Addison Mayberry, David L. Perkins, Daniel E. Holcomb

**Affiliations:** 1 Lumme Inc., Amherst, MA, United States of America; 2 Electrical and Computer Engineering, University of Massachusetts Amherst, Amherst, MA, United States of America; 3 College of Information and Computer Sciences, University of Massachusetts Amherst, Amherst, MA, United States of America; 4 Richard Cronin Aquatic Resource Center, US Fish and Wildlife Service, Sunderland, MA, United States of America; University of California Irvine, UNITED STATES

## Abstract

Miniaturized imaging devices have pushed the boundaries of point-of-care imaging, but existing mobile-phone-based imaging systems do not exploit the full potential of smart phones. This work demonstrates the use of simple imaging configurations to deliver superior image quality and the ability to handle a wide range of biological samples. Results presented in this work are from analysis of fluorescent beads under fluorescence imaging, as well as helminth eggs and freshwater mussel larvae under white light imaging. To demonstrate versatility of the systems, real time analysis and post-processing results of the sample count and sample size are presented in both still images and videos of flowing samples.

## Introduction

Traditionally, the analysis of biological samples has been restricted to a lab or a central facility. These analyses employ the use of large imaging systems such as white light microscopes, fluorescence microscopes or flow cytometers, depending on the type of sample under study. Microscopes are great for obtaining qualitative information about the cells such as size, shape, distribution, or cellular inclusions. The downside of microscope is the cost, size, and fragility of the equipment. Manual analysis using a microscope requires a trained eye for proper interpretation, making analysis time consuming. Fluorescent imaging devices screen a range of diseases including blood related disorders, cancers, and AIDS. Microscopes have been an indispensable equipment in diagnosis by providing both qualitative and quantitative information about the sample. The downside of microscopes is manual screening; the reliability of this method depends on the experience of the clinician or the pathologist. Flow cytometers are automated devices that also provide quantitative and qualitative information, making them the state-of-the-art technique to screen for human immunodeficiency virus (HIV). The drawback of these devices is the costs associated with the device; commercial flow cytometers cost $35,000 or more and have a maintenance costs of $6000 per year [1]. The other drawback of this device is clogging; analysis is performed only on one cell at a time, hence the size of the aperture must be small. The device can handle cells up to 100 *μ*m, which is wider than most cells. The problem arises when two or more cells are lumped together or when an abnormal cell is being analyzed. Cancer cells and other unhealthy cells usually tend to be larger than healthy cells, clogging the system during analysis.

Two solutions have been proposed to address the drawbacks of the device and the techniques: miniaturization and mobile phone microscopy. An overview of miniaturized subsystems that can be used to develop a miniaturized microscope is given by Helmchen [2]. The working principle of the miniaturized systems proposed by Ghosh et al. [3] is similar to a conventional lab microscope; instead of traditional excitation sources and imaging techniques, the system integrates a CMOS imager, filter cubes and mirrors, an FPGA and other electronics to reduce the size of the imaging system. Although miniaturization improves portability, it introduces new challenges and adds complexity to the overall system.

Mobile phone microscopy is the other approach that appends optics to a mobile phone camera enabling it to perform white light and fluorescence microscopy. Groups have demonstrated cell sample detection, sample count, and fluorescence detection with this approach. Diagnosis of sickle cell anemia and malaria using mobile phone microscopy has been previously published [4]. The setup used by this group consists of precisely placed lenses and filters aligned with the mobile phone’s camera. Later work employs a similar optics holder to detect fluorescence from biological sample [5]. In both the systems, images are captured using the inbuilt camera application and analyzed manually or transferred to a computer for post processing.

This paper improves on existing mobile phone microscopy techniques by detecting sample and offering quantitative and qualitative information in real time. While other published work has made significant contributions to specific aspects of mobile phone microscopy such as optics and specialized imaging components, this work focuses on developing a simple setup for a broad range of biological applications and sample detection techniques. The proposed setup would consist of a 3D printed jig to align simple optics and the sample to the mobile phone microscope. An inbuilt android application will capture images of the sample which can either be automatically analyzed in real time or sent to a computer for offline processing. In this work, we have demonstrated the capability of the application to detect and provide sample count in both stationary images and from videos of biological sample. Image processing techniques to analyze the sample in real time using Java and post processing using Matlab is also outlined in this work. To demonstrate that the proposed system is capable of handling a wide range of biological sample, this system was used to analyze three distinct biological samples: fluorescent beads, helminth eggs, and glochidia.

The system was tested with fluorescent beads to demonstrate its significance in medical applications. Existing miniaturized systems that can detect fluorescence employ techniques such as total internal reflection [6], time-domain excitation separation using high speed pixels [7], spin coated filters [8], and fiber optic phase plate with fixed sample-sensor separation [9]. Although these devices are smaller than the traditionally used flow cytometers, the complexity, fragility, and costs of these systems limits their use to laboratory settings. The ability of the proposed mobile phone based system to detect fluorescence signal drastically improves its significance in disease screening especially, at remote and resource limited areas.

Soil-transmitted helminths are among the most common infections that affect poor and deprived communities [10]. Traditional screening of helminth eggs involves analysis of a stool smear under a microscope. Microscopic analysis requires a trained technician to systematically examine the entire fecal smear. This problem has been partially addressed by replacing microscopes with mobile devices such as the demonstration by Bogoch et al. that detection of soil-transmitted helminth eggs is possible with a mobile-phone based microscope [11]. Their method requires the preparation of a fecal smear, and sample analysis is performed using the Kato-Katz technique; the reported time for Kato-Katz is 48 minutes per sample [12]. The proposed system in this paper improves on their mobile-phone microscopy by producing high quality images and reducing the cost, time, and resources required for screening. The sample analyzed with the proposed system, does not require sample preparation or the Kato-Katz method.

Glochidia are the larval stage of freshwater mussels, the most imperiled of any major group of animals in North America. Artificial propagation has been one of the approaches used to restore mussel populations and this process requires counting large quantities of glochidia. Traditionally, the counting is done by a foreman with the use of a microscope [13]. However, counting microscopic glochidia is labor intensive and prone to human error. We explore how glochidia counting can be automated by use of image processing. Some previous studies have been done on the counting problem of larger rigid objects such as cars or pedestrians [14] [15] which do not change their shapes and sizes, and are easily visible with the naked eye and therefore less challenging to acquire in images for processing. The problem of glochidia counting is more complex because the objects of interest are microscopic, have variations in size and shape, and can resemble other organisms and particles in the sample.

The next section describes the materials and methods involved in the imaging set up, techniques used to prepare the sample, and imaging techniques that were employed for data analysis. Section 3 discusses the results from data analysis and compares the results from the 3 techniques: manual analysis, real time analysis, and analysis using Matlab.

## Materials and methods

In this section we describe the setup used for our experiments and the device fabrication techniques used to handle the problem. More specifically, we will discuss the following:

- Imaging setup— Optics, jig design, microfluidic device design.
- Sample preparation— fluorescence beads (medical applications), helminth eggs (diagnostics), and mussels (counting and inoculation).
- Imaging techniques— Manual analysis, Post processing (OpenCV) [16], Real-time processing (Android platform)

### Imaging setup

Mobile phones are widely available and most devices have an inbuilt camera with multi-megapixel resolution. The camera on the phone can be used in macro mode by placing a simple ball lens in front of it. The resulting magnification can be increased by simply picking a ball lens with a different focal length. Data for this work was collected from an LG Nexus 5 phone. For data collection, two setups were employed: one for collecting data from stationary samples and one for collecting data from samples inside a microfluidic channel. The analyses performed on the samples are summarized in Table 1.

**Table 1:** Overview of samples and techniques used.

Sample	Device	Detection	Measurement
Helminth	flowing (video)	Color segmentation / Edge detection	count
Glochidia	flowing (video)	Background subtraction / Edge detection	count; size
Flourescent beads	still (image)	Watershed Algorithm / Edge detection	count

**Fig 1:**
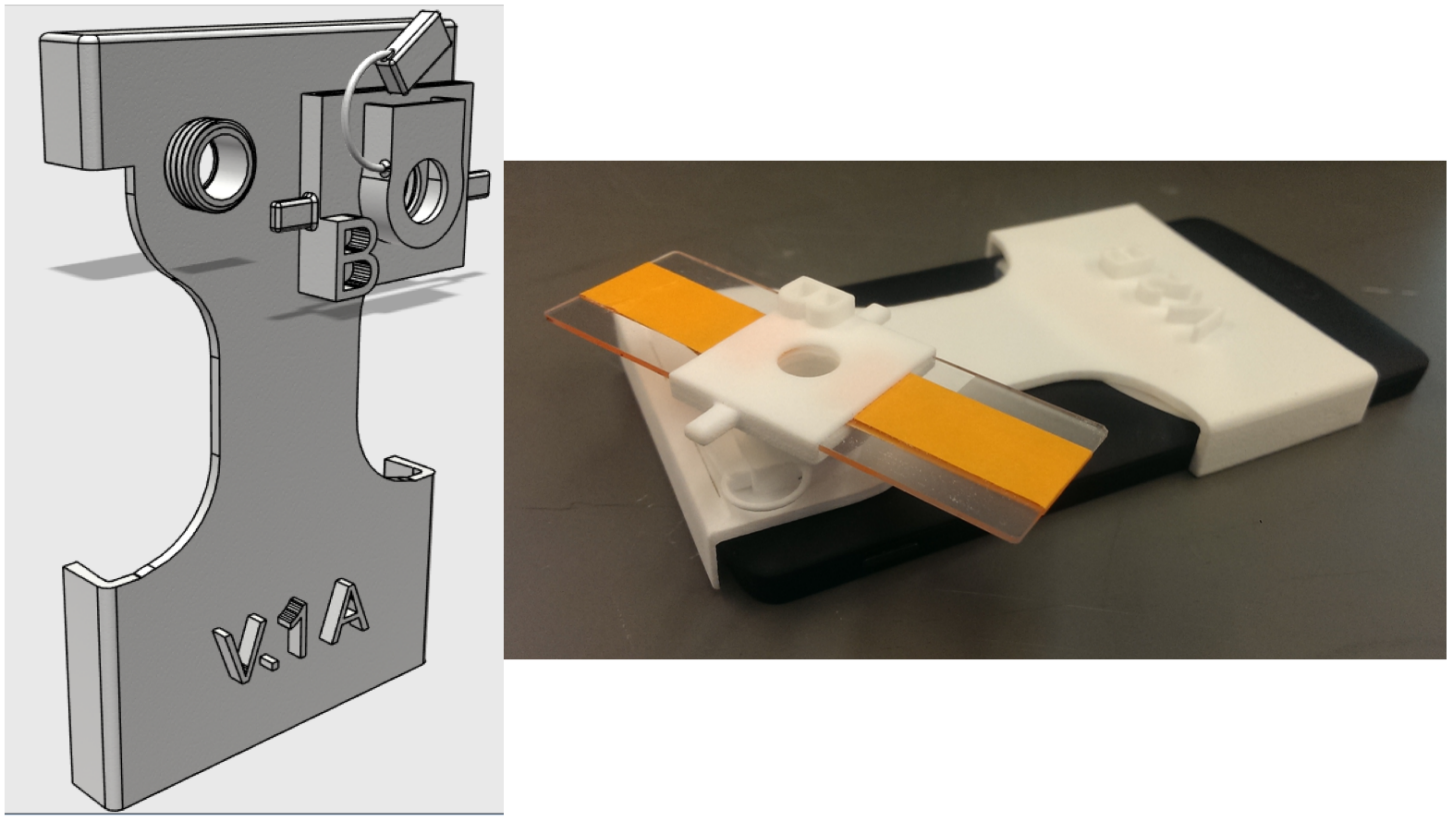
3D printed jig used in mobile phone microscopy to analyze stationary samples.

The setup shown in Fig 1 was employed to collect data from stationary sample, i.e the sample was placed on a glass slide and protected with a cover slip. The imaging set up consists of a socket for the ball lens to be placed above the phone’s camera, a slot for the glass slide, and a second socket for a filter if fluorescence imaging is used. The slide slot has threads to keep the slide in place and allows adjustment of the sample sensor separation. The jig was designed in AutoCAD and 3D printed.

**Fig 2:**
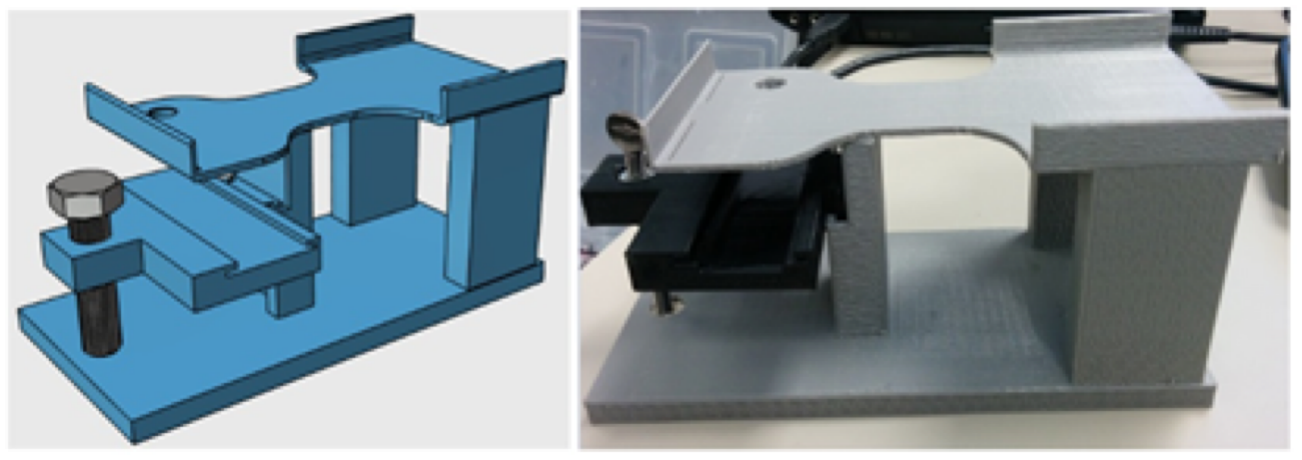
3D printed jig used in mobile phone microscopy to analyze samples in microfluidic devices.

Although most biological samples are analyzed after they are fixed to a surface, there are many benefits to analyzing flowing samples. The primary benefit is reduction in analysis time; throughput of sample analysis can be drastically increased by analyzing flowing samples. To achieve this with mobile phone microscopy, a special jig was designed to interface microfluidic devices with mobile phones. The illustration and the printed prototype of the jig is shown in Fig 2. This jig has a base similar to the jig used to analyze stationary samples and is fitted with a holder for the microfluidic device. The sample sensor separation can be changed with the help of the metal knob shown in the Fig 2.

**Fig 3:**
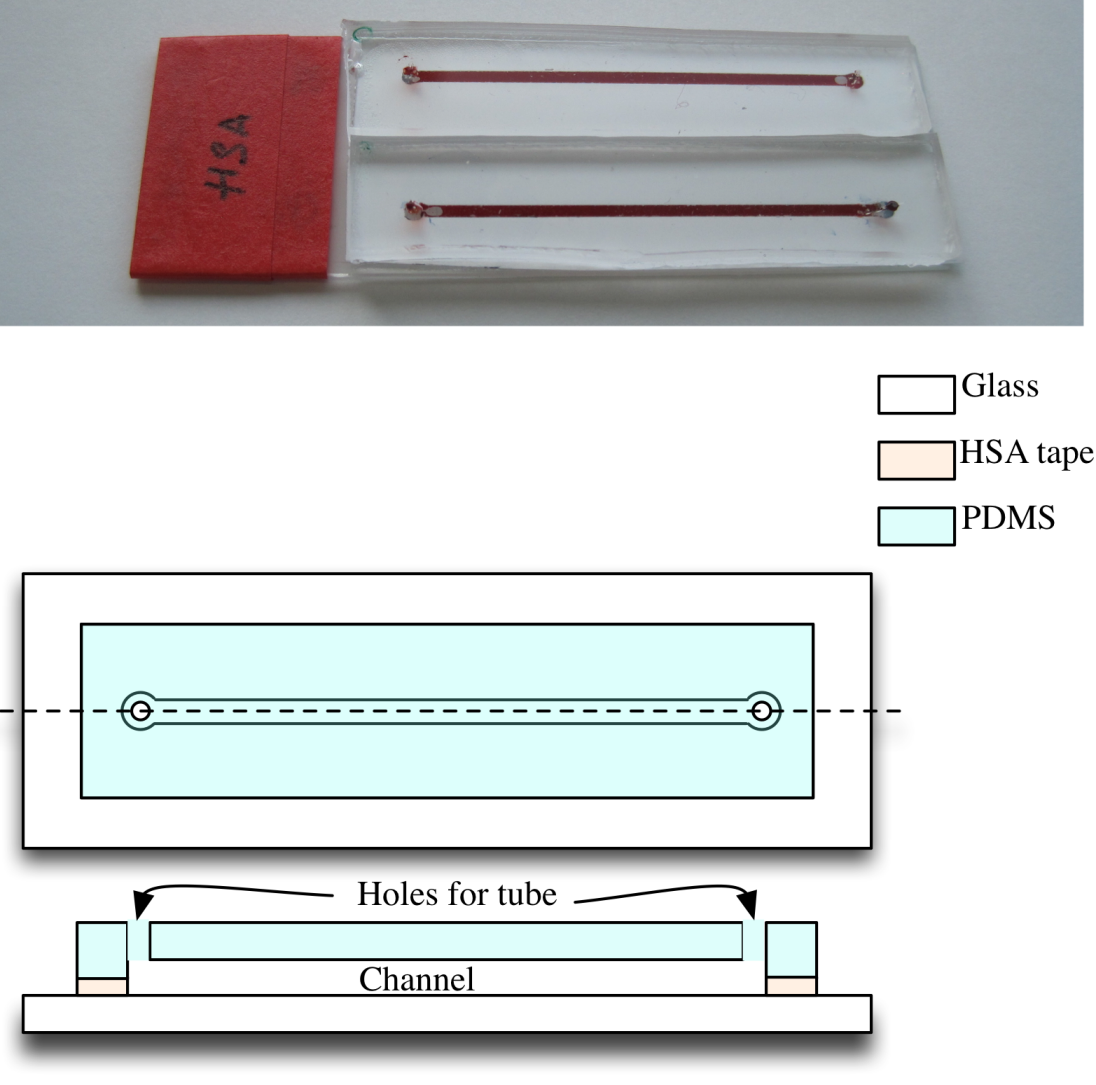
Microfluidic device used for still sample experiment.

In this work, for still samples, the microfluidic networks, shown in Fig 3, were fabricated on a microscope slide by patterning heat sensitive adhesive tape (HSA) and sealing the device with a slab of cured Polydimethylsiloxane (PDMS). A simple network with a straight channel and ports was designed for this experiment. The tape was patterned and layered to obtain a net thickness and channel width of 200 *μ*m and 1 mm respectively. Most traditional microfluidic systems are made out of PDMS using a technique known as soft lithography. This requires a master mold to fabricate the devices. The advantage of using tape based devices is the flexibility to change the design of the microfluidic device without the need for a new master mold. Although simple straight channel microfluidic system was employed in this work, any complex network of systems can be interfaced with this set up for analysis.

**Fig 4:**
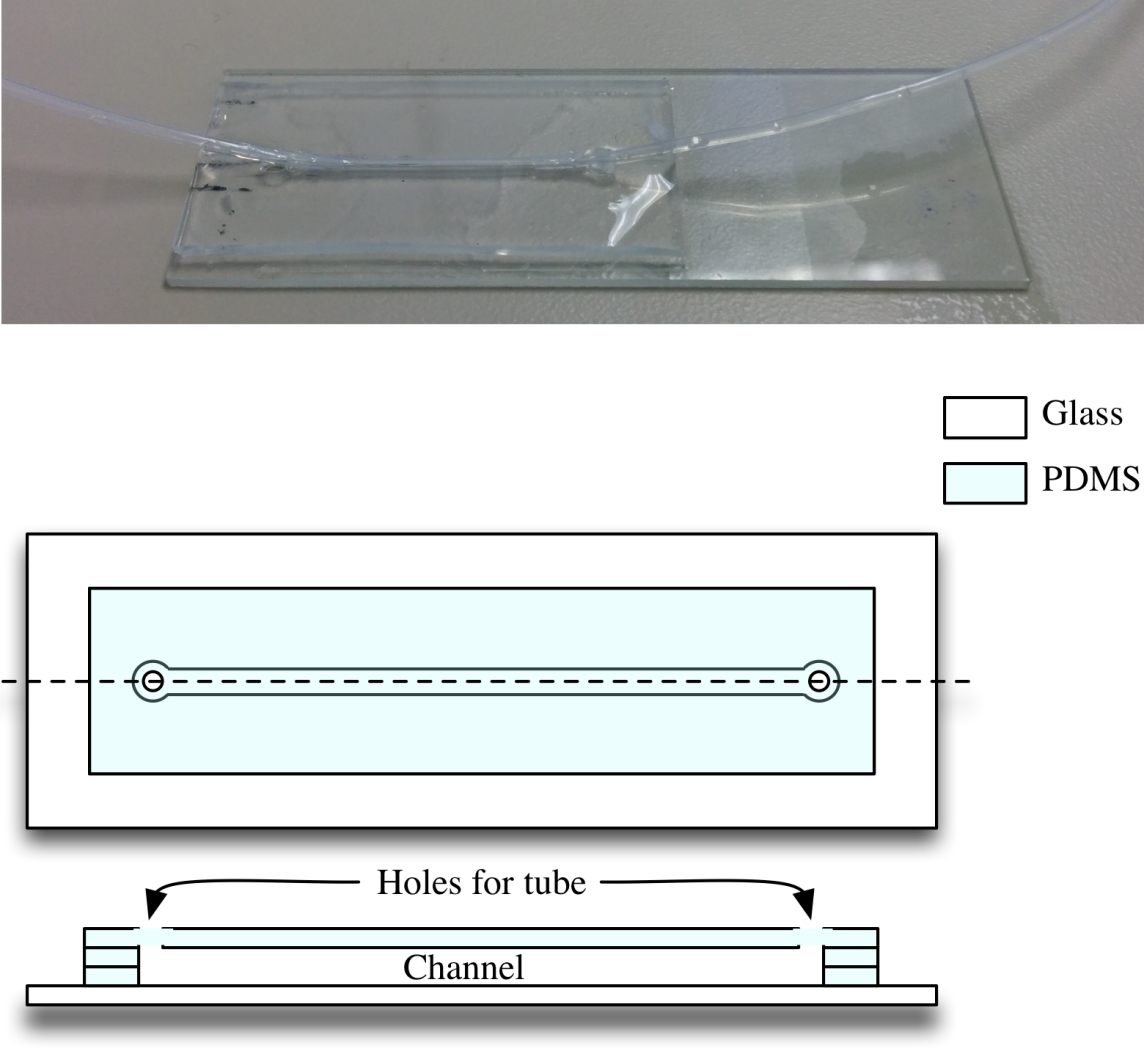
Microfluidic device used for flowing sample experiments.

For the counting experiments which involved flowing samples, the microfluidic device was fabricated by using sheets of PDMS with a thickness of 250 *μ*m. The sheets were cut using a Silhouette Cameo desktop cutter to form the desired channel shape. Multiple layers of the cut sheets were stacked on top of each other to attain the desired channel height. A plasma cleaner is used to bond the PDMS layers to each other. A final PDMS layer facilitating holes for tube insertion for flowing the sample inside the channel is placed on top. Tubes are inserted at both ends to provide sample input and output through the channel. The tubes are sealed using a mixture of PDMS and silicone elastomer curing agent in the ratio 10:1. The tubes are carefully inserted horizontally instead of vertically to limit clogging at the insertion point, which was found to be a problem with vertical insertion due to presence of large debris particles in the sample. The final device is shown in Fig 4.

### Sample preparation

In this work, three different biological samples were used to demonstrate the robustness of the proposed imaging system. The samples being imaged are fluorescent beads, helminth eggs, and glochidia. Sample preparation techniques and handling methods are listed in this section.

To demonstrate that the system can detect fluorescence, commercially available fluorescent beads (Spherotech FP-10045-2) were used. These beads are commonly used to calibrate clinical flow cytometers. During imaging, 1 *μ*L of the bead solution was mixed with 500 *μ*L of deionized water to make the working solution.

To demonstrate the capability of detecting helminth eggs, stool samples from five mice infected with T. muris (whipworm) were collected. The feces samples were diluted by mixing one gram with 30 mL water and stored at 4°.

For propagation of freshwater mussels, glochidia are extracted from gravid females and used to either inoculate host fish, on which they transform into juveniles, or put into petri dishes for growth and development in vitro. To create samples used in this work, glochidia were pipetted into in clean water, with care given toward minimization of extraneous debris. The target concentration for sample collection is approximately 50 glochidia per 5mL of water.

### Imaging techniques

To develop an algorithm for counts we first begin with detection and counting of fluorescent beads.

#### Counting fluorescent objects

**Fig 5:**
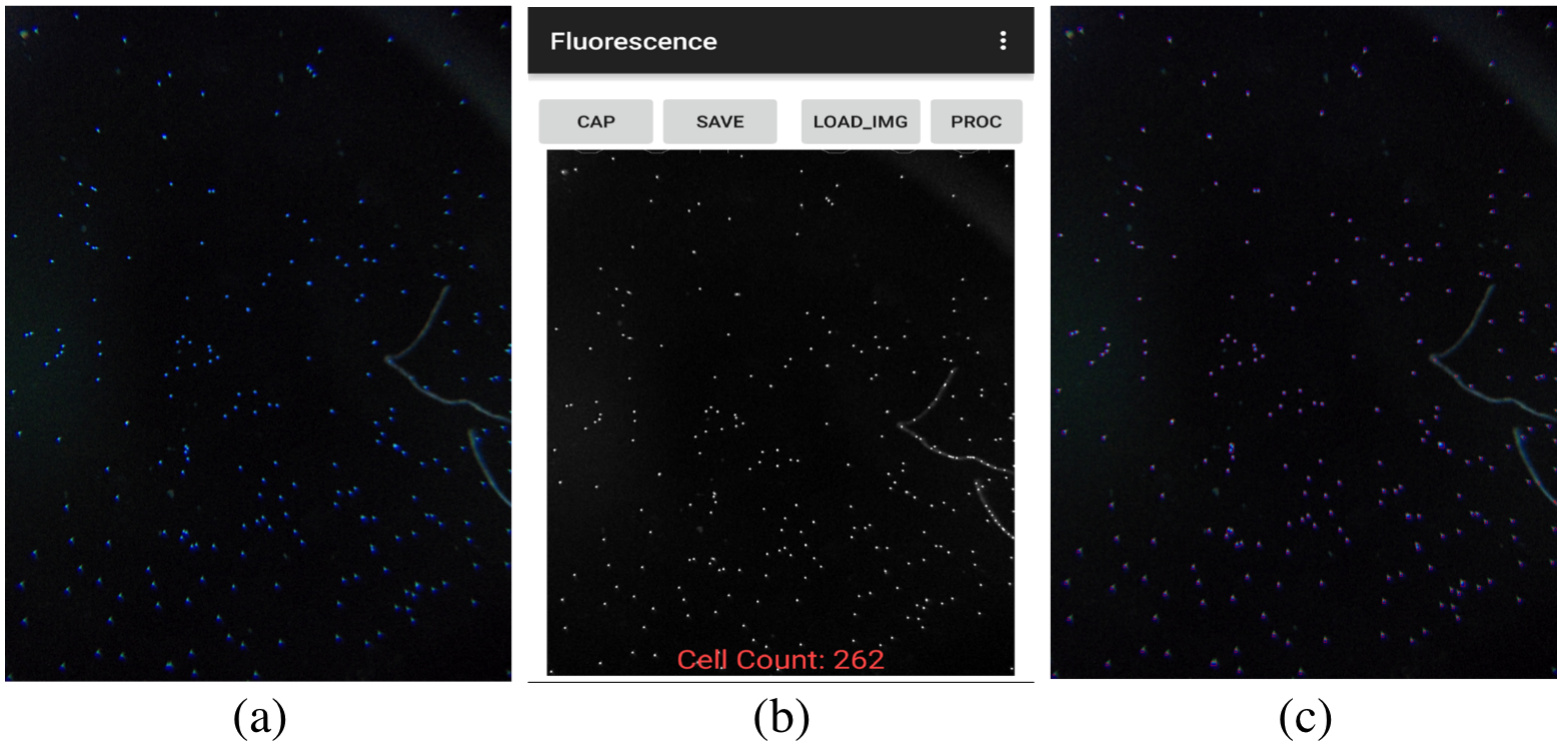
Figure showing the processing of fluorescent beads on two platforms. a) Original image of fluorescent beads. b) Processed image on Android showing count. c)Processed Image on PC.

The fluorescent-object-count scenario is somewhat simpler than the helminth and glochidia scenarios, due to the fact that it is taking still images as input instead of video and also because the contrast between target objects and background is much higher, see Fig 5a. However, the algorithm must still be designed carefully to deal with visual artifacts such as blurring due to the relatively low quality of a smartphone camera and also to be able to properly count objects that have clumped together and may appear as one single, large object if not processed carefully.

Our algorithm is derived from the watershed computer vision algorithm for identifying regions of high contrast in an image [17], using only the portions relevant for our task and with some additional filtering added. Intuitively, this algorithm identifies regions that are potentially the center of a fluorescent object, then combines those regions in a carefully controlled way to determine which are actually separate objects for counting.

We first preprocess the image using three standard computer vision techniques— top-hat transform, histogram equalization, and median blur— to ensure that the contrast is as high as possible and that illumination is consistent across the entire image. From there, we identify candidate object centers using simple thresholding and perform a “distance transform” which assigns every pixel in the image to the closest candidate center, which creates clusters of pixels representing candidate objects. At that point it is simply a matter of shrinking the regions using an “erode” operation so as to distinguish neighboring objects. Once that is complete, the objects can easily be counted using connected-components techniques.

#### Counting helminth eggs and glochidia

**Fig 6:**
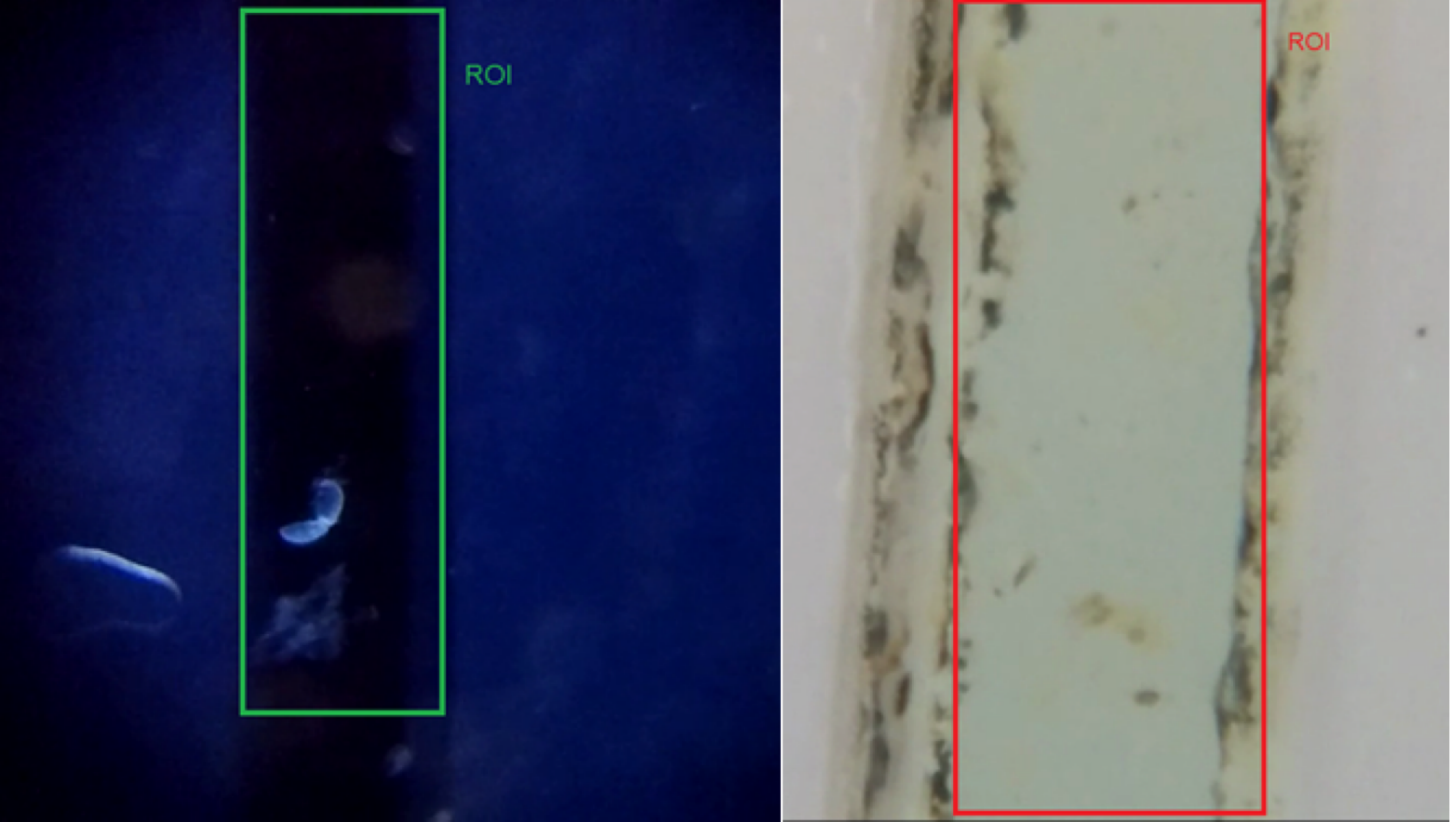
Rectangles show regions of interest in videos for glochidia (left) and helminth egg (right) experiments.

The first step in processing the sample is to crop the video to the region of interest to reduce the size of the video, thereby making the computation easier on the mobile computing platform. The region of interest (ROI) in our case is the width of the channel through which the sample is flowing. Fig 6 shows the channels and ROIs for both the helminth eggs and glochidia.

**Fig 7:**
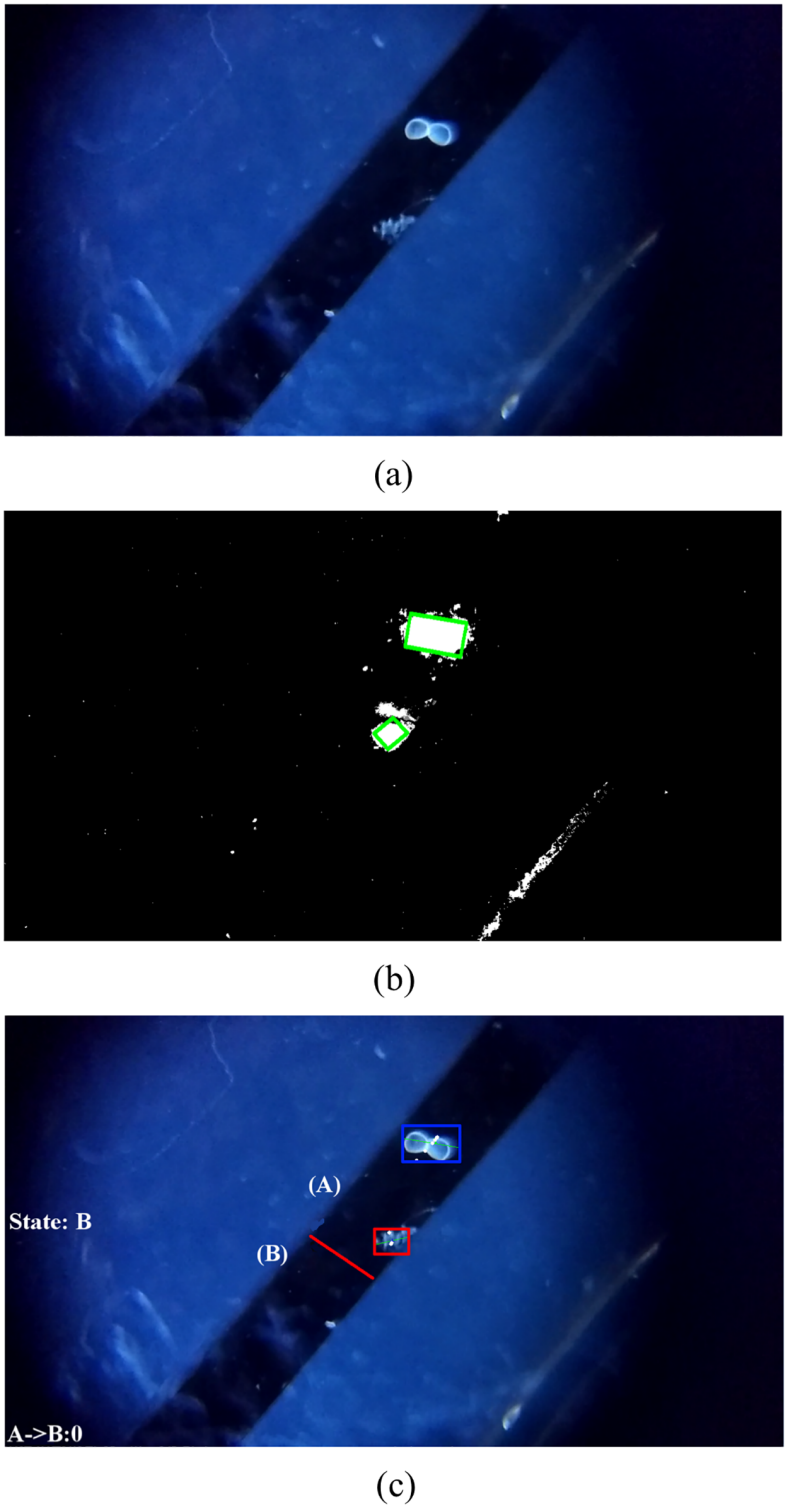
Figure showing the various processing steps involved in the processing of glochidia sample. a) Original image of microfluidic channel. b) Result of Background subtraction, morphological operation and contour detection on frame. c) Blob tracking and counting using virtual line.

**Fig 8:**
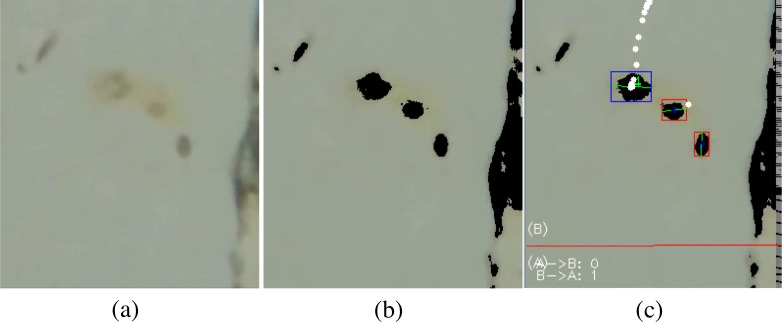
Figure showing the various processing steps involved in the processing of helminth eggs sample. a) Original image of microfluidic channel with Helminth eggs. b) Processed video showing detected blobs after Color segmentation, Background subtraction and morphological operation on frame. c) Blob tracking and counting using virtual line.

Helminth eggs and glochidia are processed using the same procedure, except that an additional pre-processing step of color segmentation is applied to the Helminth videos due to poor contrast between the Helminth eggs and the background (compare Figs 7a and 8a), as well as the larger amount of random moving particles in the Helminth sample. The color segmentation step partitions the video into regions based on changes in color, and this converts the low contrast observed in Fig 8a into the higher contrast observed in Fig 8b. Color segmentation was not needed in glochidia because the samples already have higher contrast against the channel which has minimal debris particles (see Fig 7a). After applying color segmentation to Helminth eggs, the subsequent processing steps applied to Helminth eggs and glochidia are the same.

The next step is to do segmentation to separate out the object of interest from the background. Background subtraction (BS) is a common and widely used technique for stationary video sources that generates a foreground mask that isolates the pixels of moving objects in the video. Background subtraction calculates the absolute difference between the current frame and a background model to produce the foreground mask. The background model contains the stationary or non-moving part of the video, which are all the parts that would be considered as background. Background modelling consists of two steps namely Background Initialization and Background Update. The first step takes the initial frame as the background model, and the second step is performed recursively to adapt to the changes in the background (e.g. lighting, camera position, inserted background objects, etc) [18]. We are using the Pixel-Based Adaptive Segmenter (PBAS) technique from [19] to perform background subtraction. The reason for using this technique is its low computation complexity. This algorithm is much faster but less accurate than the common Mix of Gaussian (MOG) subtraction technique. Fig 7a shows the original image of the channel and Fig 7b shows the foreground mask generated after performing background subtraction on glochidia sample. The white blob in Fig 7b is formed due to presence of glochidia moving through the channel. This blob would then be used for tracking and calculating the centroid of the glochidia.

After creating the blobs of objects, Morphological operations are performed on the image to find objects having a shape that matches the target. The type of the structuring element used depends on the size and shape of the target object. For glochidia an elliptic structuring element, that is, a filled ellipse inscribed into the rectangle is used. For Helminth eggs, we use the rectangular structuring element. A series of erosion and dilation morphological operations are applied to the frame to remove noise in the video after applying Background subtraction. This also closes the broken blobs which helps in their detection as one object. Canny edge detection [20] followed by contour detection is performed. Fig 7b shows the result of these operations performed on a background subtracted frame.

The contours of the objects are apparent after background subtraction. From the contours, the size of the objects and also the number of objects in the scene can be calculated. These objects are then filtered based on their area to eliminate objects that are too small or large to be the targets. Fig 7b shows the labeled blobs after the operations discussed above; only the moving blobs have been marked (by green boxes) in the image.

To enable counting, the objects that survive the filtering operation are then given identifiers and are tracked in the video using the cvBlob library in openCV. To track the objects, the centroid of each blob is calculated in the starting frame. In the next frame, the centroid of the blobs are calculated again and based on the position of the centroid, the blob IDs from the previous frame are assigned to nearest blob in the next frame. This process is performed repeatedly to keep track of blobs within the region of interest. When a tracked object’s centroid crosses a virtual line in the ROI (shown in red in Figs 7c and 8c), a check is performed to see whether the crossing object matches the target object. If the match is detected the count of the object is incremented.

#### Measuring glochidia sizes

**Fig 9:**
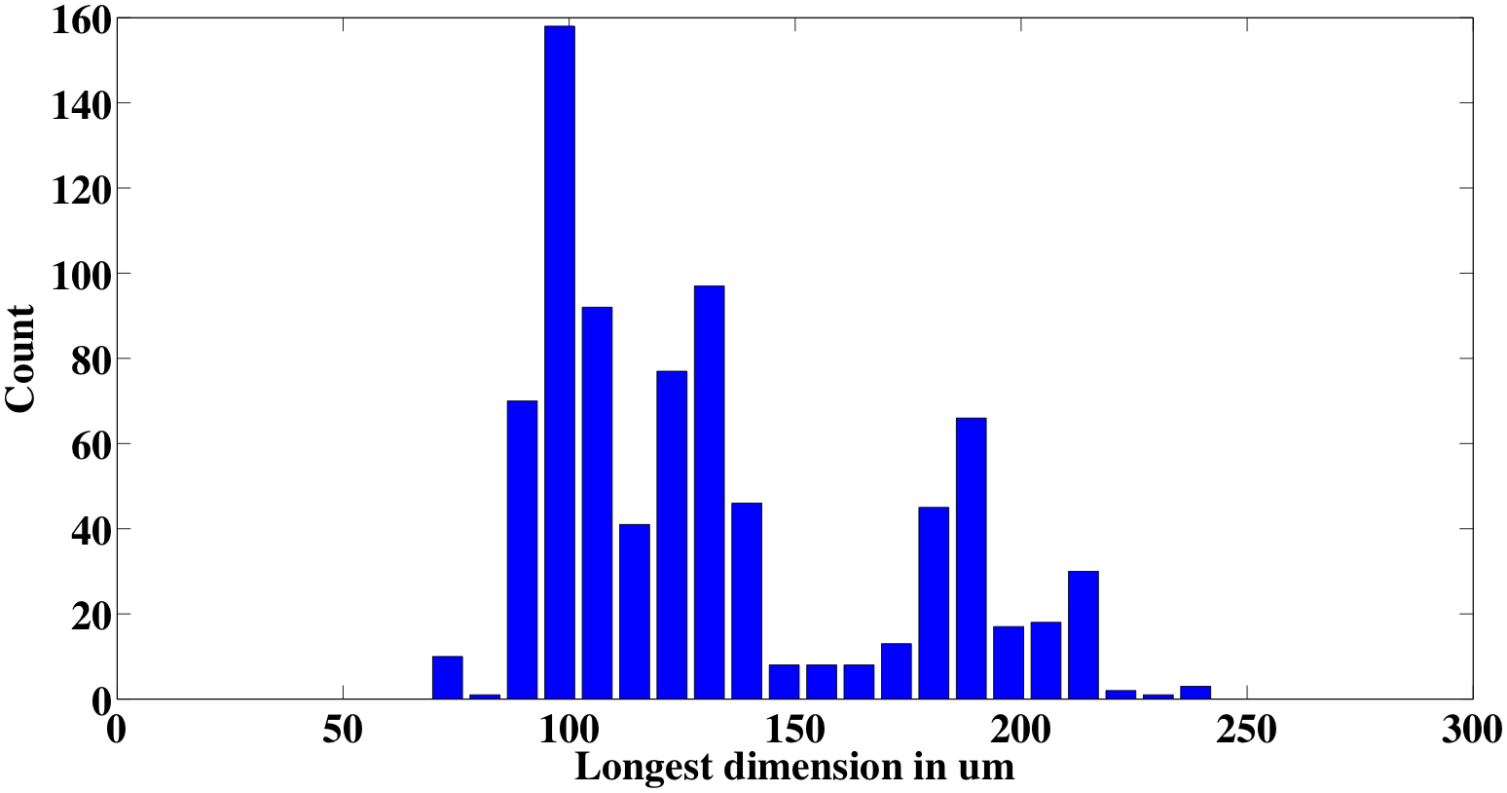
Glochidia size distribution histogram.

**Fig 10:**
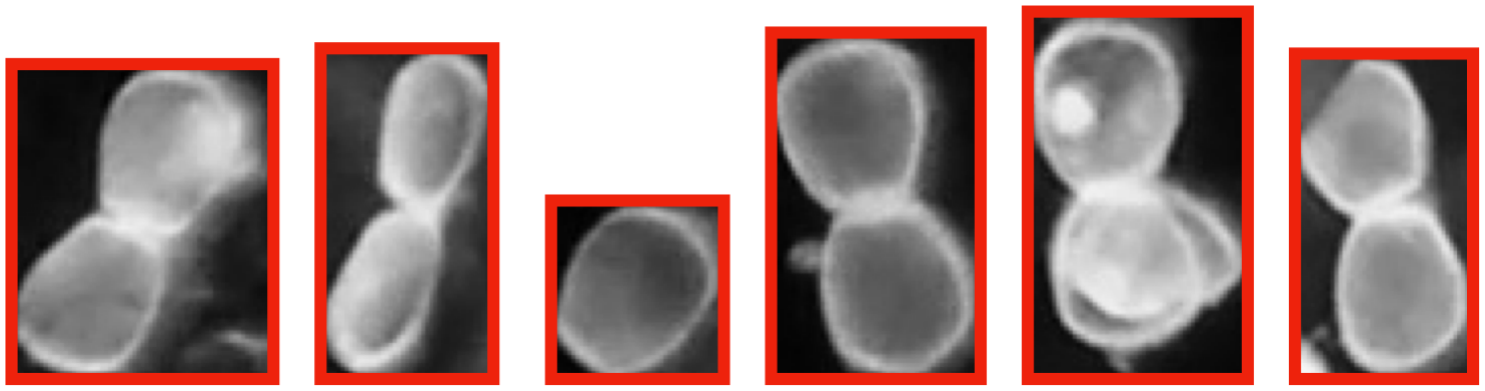
Example of the bounding boxes used to measure glochidia sizes.

To determine glochidia size, automated measurements are also performed after counting. To measure the size, the known channel width of 350 *μ*m is used to calibrate a pixels-to-length conversion factor that translates the size in number of pixels to size in standard length units. The longest dimension of the bounding box of the glochidia blob is used as its size measurement. Fig 9 shows the histogram of glochidia sizes for a count of 400. Fig 10 shows the bounding boxes that were generated when measuring the sizes of six different glochidia; in most cases, the bounding boxes are found to match tightly to the glochidia dimensions, giving confidence that the measurements are measuring the glochidia appropriately. Note that the image processing recognizes and measures the glochidia without making any distinction about whether their shells are open or closed (see Fig 10); the mixture of open and closed shells leads to a bimodal distribution in the measured sizes.

### System setup

**Fig 11:**
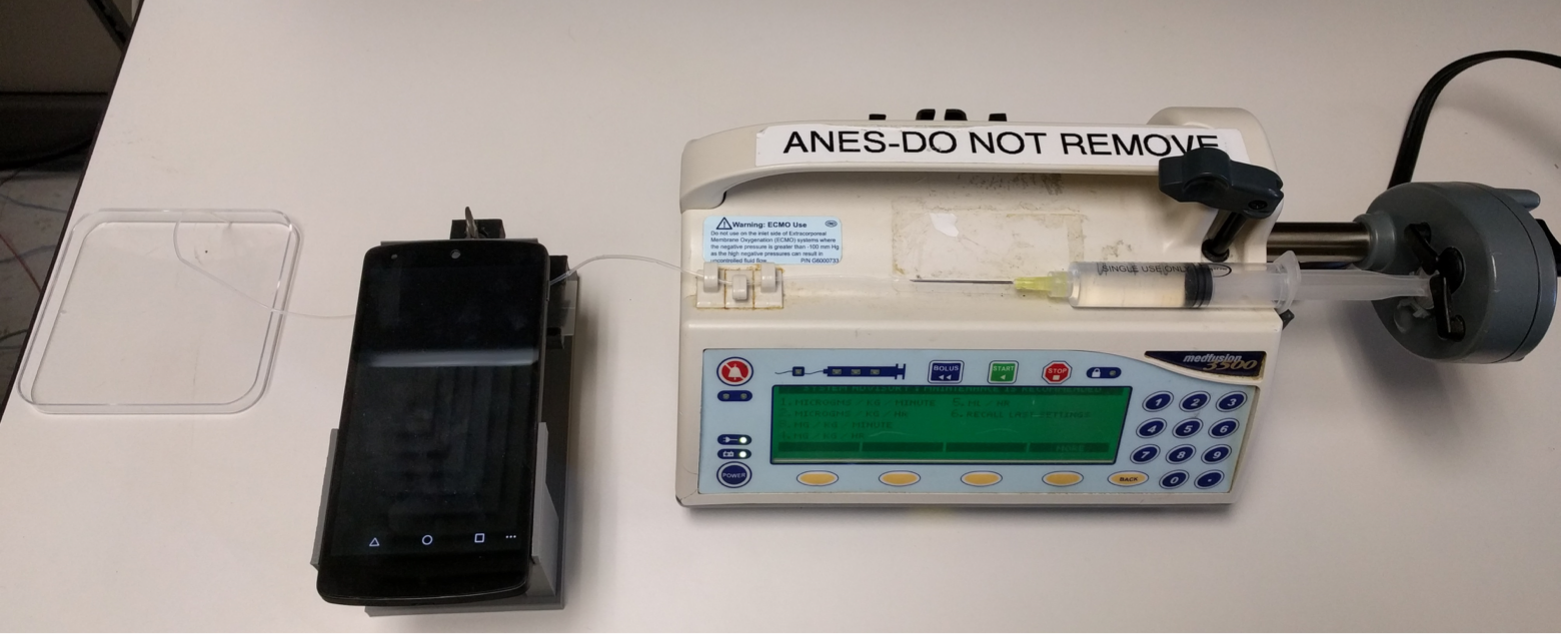
System setup to automate sample counting process using syringe pump, mobile phone on printed jig, and container to catch the samples after processing.

Fig 11 shows the setup used to automate the glochidia counts. The syringe is filled with the sample and is attached to a automated dose delivery system. The tube from the microfluidic channel is connected to the syringe and the sample is delivered automatically at a rate of 60mL/hour. The Android phone captures the video and performs the processing to extract counts and sizes. Although the syringe pump is used in these experiments for consistency, a manual hand-operated syringe could be used in place of the pump for experiments done in the field.

## Evaluation

**Fig 12:**
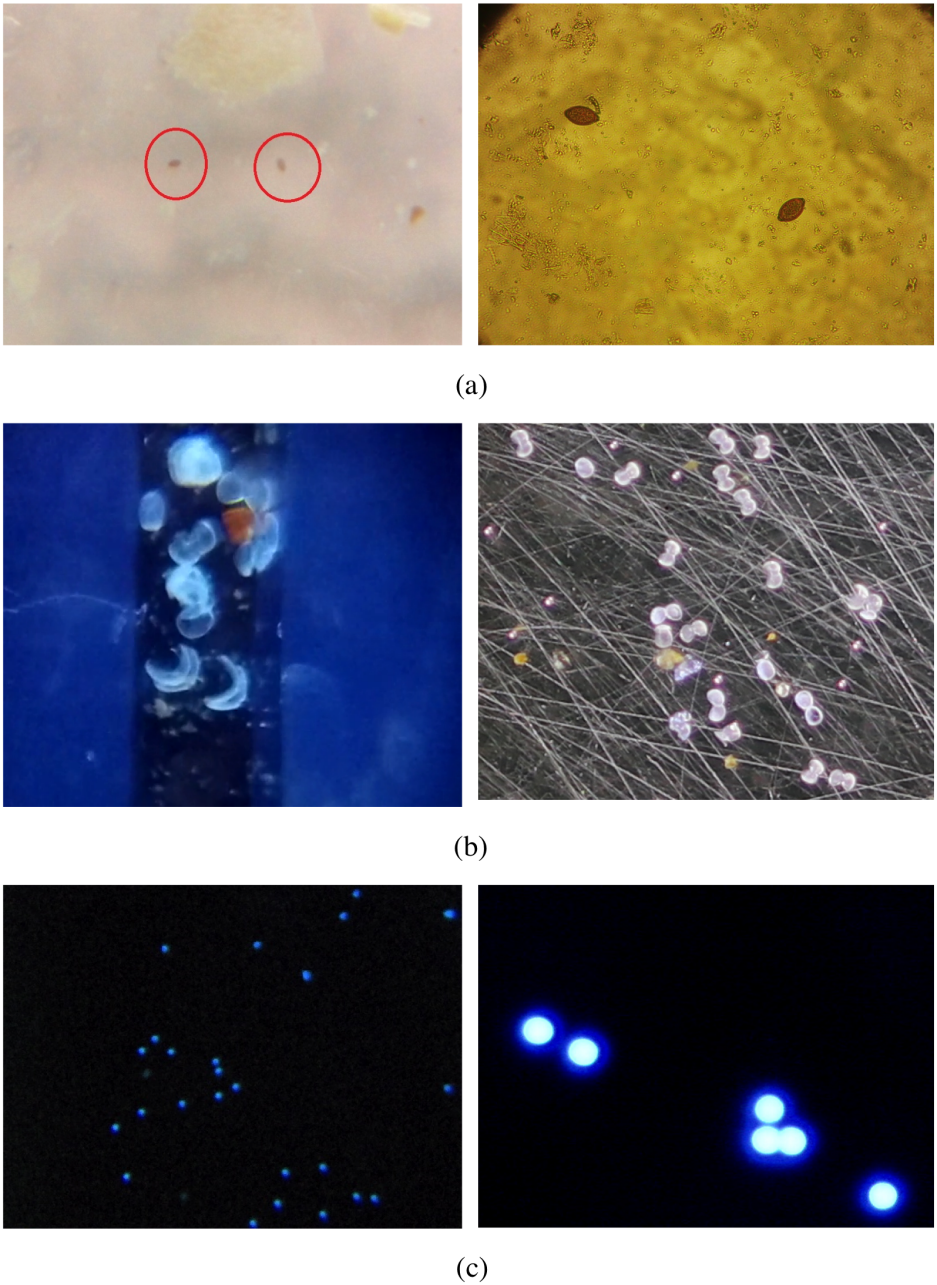
Comparison of image quality using a traditional microscope (left) and mobile phone microscope (right). Traditional microscope images of the a) eggs, b) glochidia, and c) beads were captured under 10X, 20X, and 20X respectively.

In this section we discuss the pros and cons of using the mobile phone microscope and compare the technique with the traditional bench top lab microscopes. Fig 12 shows the image comparison of the three samples under consideration. The images shown on the left were taken with a lab microscope. We can clearly see the superior quality of the images taken by the lab microscope. The increase in the image quality comes with a substantial increase in cost as well as restriction in mobility of the setup. This improvement is not worth the cost and restriction of the setup to lab environment.

The mobile phone microscope can be carried to any location and nowadays even inexpensive mobile phone costing under a hundred dollars have good cameras. In light of this, one may conclude that automated detection using mobile phone microscope is superior to traditional microscope in settings where cost and mobility are important.

## Results

**Fig 13:**
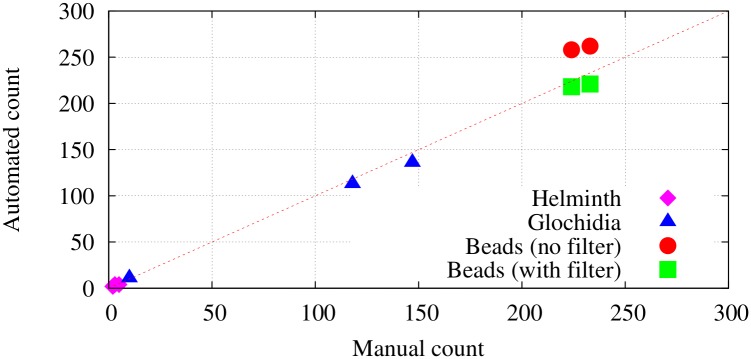
Correspondence between manual counts and automated counts on the different samples.

The results of counting experiments on the mobile phone microscope platform are found to be competitive to the results obtained by manual counting, as summarized in Fig 13. Table 2 shows the counts obtained for two samples of fluorescent bead using manual counting, automated counting performed in software running on the Android phone, and automated counting with an additional filtering step that pre-processes images to remove pixels falling outside of the expected range of values. Table 3 shows the counts obtained from three different helminth samples and three glochidia samples. The samples were processed under constrained lighting conditions. A LED light source was attached closely to the side of the PDMS device to provide bright lighting to allow the use of low exposure setting to prevent motion blurring and ghosting. Manual count was performed on the recorded video and a comparison was made with the reported software count. The mean absolute percentage error across all sample counting experiments is 11%.

**Table 2:** Fluorescent beads count results.

Sample	Actual Count (Manual)	Software Count (no filter)	Software Count (with filter)
1	223	262	221
2	224	258	218

**Table 3:** Comparison of manual and automated counts from helminth and glochidia video samples captured on smart phone camera.

Video Sample	Actual Count (Manual)	Software Count
Helminth 1	3	4
Helminth 2	5	4
Helminth 3	2	2
Glochidia 1	10	11
Glochidia 2	113	118
Glochidia 3	136	147

## Conclusion

This work has demonstrated the feasibility of mobile phone microscopy as a simple platform for performing real-time analysis on important biological samples. The proposed system could not only enable screening of soil-transmitted helminths in resource limited areas but would also increase the throughput of screening when compared to traditional methods. The techniques show promise for automated glochidia counts during propagation, but practical application is hindered by low glochidia density needed for accurate counts and low flow rates needed to prevent clogging. Detection of fluorescence in sample enables point-of-care diagnosis for a wide range of diseases and disorders. Overall, the ability of the proposed system to perform fluorescence imaging, detect flowing samples, and determine size of the sample has numerous applications in medicine, biology, environmental studies, microfluidics, and disease screening.
